# Supratrigonal cystectomy and augmentation cystoplasty with ileum or ileocecum in the treatment of ulcerative interstitial cystitis/bladder pain syndrome: a 14-year follow-up

**DOI:** 10.1007/s00192-022-05110-y

**Published:** 2022-03-01

**Authors:** Fabian Queissert, Benedict Bruecher, Arndt van Ophoven, Andres J. Schrader

**Affiliations:** 1 Department of Urology and Pediatric Urology, University Hospital of Muenster, Albert Schweitzer Campus 1, 48149 Muenster, Germany; 2 Department of Neurourology, Marienhospital Herne, Herne, Germany

**Keywords:** IC/BPS, Ulcerative interstitial cystitis, Supratrigonal cystectomy, Augmentation cystoplasty, Primary bladder pain syndrome

## Abstract

**Introduction and hypothesis:**

This study analyzes the long-term results of supratrigonal cystectomy and augmentation cystoplasty in patients with severe ulcerative interstitial cystitis/bladder pain syndrome (IC/BPS) and reduced bladder capacity.

**Methods:**

Outcome data were retrospectively and prospectively collected and analyzed in women who underwent supratrigonal cystectomy and augmentation cystoplasty for ulcerative IC/BPS at Muenster University Hospital between 1991 and 2006. We used cross-tabulation and Pearson’s Chi-squared test to examine how outcome is influenced by age, preoperative functional bladder volume, and choice of augmentation material.

**Results:**

After a median 171-month follow-up, analysis could be done in 26 of 27 patients. Persistent pain necessitated early revision in 2 patients (7.7%). Mean postoperative O’Leary Sant IC Score was 12.7 in the prospectively questioned patients. Responses to Patient Global Impression of Improvement (PGI-I) were: “very much better” in 15 cases (65.2%) and “much better” in 7 (30.4%). Twelve patients (52.2%) emptied their augmented bladder voluntarily, whereas 7 (32%) needed intermittent self-catheterization (ISC). The rate of patients requiring ISC tended to be lower when detubularized ileocecal bowel was used. All 5 patients (19.2%) with late relapse of ulcerative IC/BPS needed ISC.

**Conclusions:**

Severe ulcerative IC/BPS can be curatively treated in some patients by supratrigonal cystectomy and augmentation, which is associated with a high satisfaction rate and few long-term complications even over a very long follow-up. In our analysis, the need for ISC is a risk factor for late relapse, although ileocecal augmentation could increase the proportion of patients with sufficient voluntary micturition.

## Introduction

Interstitial cystitis/bladder pain syndrome (IC/BPS) is a chronic, potentially debilitating condition characterized by pain perceived to be related to the bladder in conjunction with lower urinary tract symptoms (painful urgency, pollakiuria, and nocturia) and includes a wide variety of clinical phenotypes with diverse etiologies [1]. Ulcerative IC/BPS is one clear phenotype that has implications for diagnosis, prognosis, and treatment strategies, and thus should be distinguished from other IC/BPS phenotypes [2]. This subtype seen in 10% of patients with IC/BPS can lead to severely reduced bladder capacity if insufficiently treated over a longer period of time [3]. Conservative treatment modalities such as hydrodistension and fulguration/coagulation usually achieve only a temporary reduction of symptoms [1]. Bladder removal surgery, however, offers a curative treatment approach. After initial use of incontinent urinary diversion, Garrelts first described successful substitution cystoplasty in 13 patients with classic IC [4]. A current review has demonstrated improvement or cure in 70.7% of patients with supratrigonal cystectomy and augmentation over a mean follow-up of 45.5 months (range 9–93.9) [5]. Patients with typical onset in the 4th decade of life [1] and benign primary disease have a long life expectancy. Therefore, surgical treatment for ulcerative IC/BPS should aim for long-term efficacy and safety. Good first results from our Münster cohort after this procedure and a follow-up period of 57 months were published in 2002 [6]. Owing to its very long follow-up period, our study now offers a long-term perspective on the outcome of supratrigonal cystectomy and augmentation.

## Materials and methods

A retrospective analysis was performed and supplemented by a prospective survey in female patients who underwent supratrigonal cystectomy and augmentation for ulcerative IC/BPS in the Department of Urology at Münster University Hospital between 1991 and 2006. Baseline characteristics were collected and included age at surgery and comorbidities. Preoperative data also included a bladder diary, which objectified voiding frequency day/night and the functional bladder volume. A urologist experienced in dealing with IC/BPS made the diagnosis in conjunction with urethrocystoscopy and hydrodistension under anesthesia in all cases. Validated patient-reported outcome measurements (PROMs; IC-O’Leary Score, PGI) were not available preoperatively and have been collected at the end of follow-up only.

**Fig. 1:**
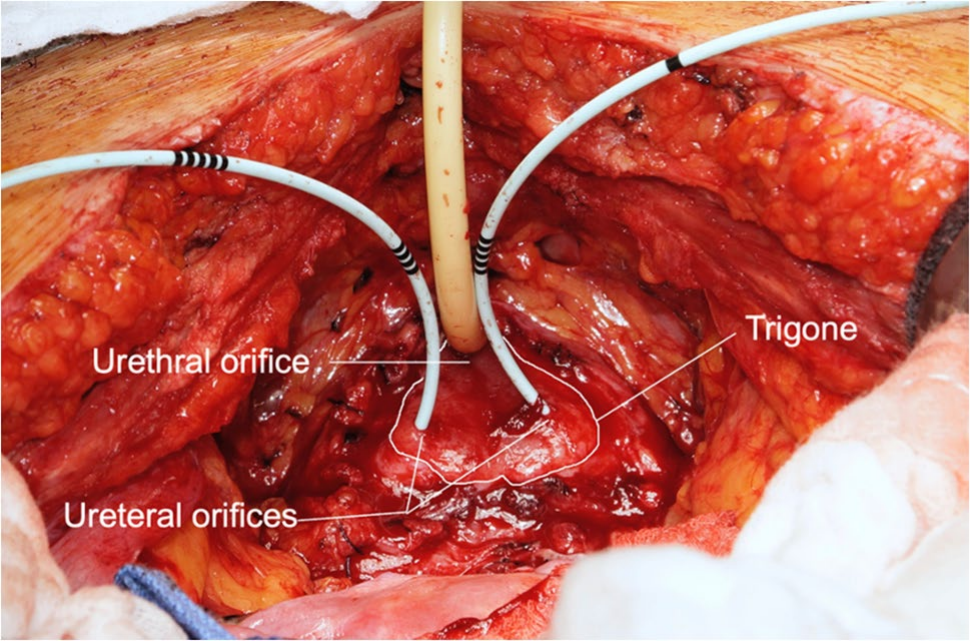
Situs with excised detrusor and residual trigone

The intervention was performed by the same surgeon. Supratrigonal cystectomy was performed in all cases, and the butterfly-shaped resection line around the trigone left an approximately 0.5- to 1-cm margin (Fig. 1).

Augmentation was performed by detubularizing an approximately 40-cm subterminal ileal segment or the cecum with an approximately 20-cm terminal ileal segment (Mainz pouch) and reanastomosing it to the trigone as a cap or w-pouch [7, 8].

Ethics approval was obtained (General Medical Council of Westphalia-Lippe; 2020-669-f-N). Descriptive analysis was used for both record-based and, when possible, prospectively acquired evaluations of complications, pain, satisfaction with the operation, the PGI-I, as well as the O’Leary Sant IC symptom and problem index, and 72-h bladder diary.

The software SPSS for Windows (version 25.0) was used for the statistical analysis. The outcomes of ileal versus ileocecal augmentation and age were examined for correlations using cross-tabulation and Pearson’s Chi-squared test.

## Results

The intervention was performed in 27 female patients with a mean age of 51.1 years (range: 23–67 years) at the time of surgery. All patients were refractory to less invasive modalities such as oral amitriptyline and/or pentosan polysulfate sodium (PPS), electromotive drug administration (EMDA), intravesical instillation of glycosaminoglycans and dimethyl sulfoxide (DMSO), hydrodistension, and/or fulguration of ulcers. Preoperative bladder diaries identified a mean diuria of 25.3 (range: 14–40) and a mean nocturia of 8.1 (range: 2–15, Table 1). The mean functional bladder volume, measured by micturition protocol, was 64.5 ml. Histologically, severe ulcerative IC/BPS and reduced bladder capacity were shown in all cases. Augmentation was performed using a detubularized ileal segment in 8 (29.6%) and an ileocecal pouch in 19 (70.4%) patients. Unilateral ureteral reimplantation was performed concomitantly in two cases and Burch colposuspension in one case.

**Table 1:** Baseline characteristics and postoperative results after supratrigonal cystectomy and augmentation cystoplasty (± standard deviation (SD)

	All	Ileal augmentation	Ileocecal augmentation	*p*
Baseline characteristics				
Cases, *n*	26	8	18	
Age at surgery, years	51.1 (±13.3)	47.5 (±13.7)	52.7 (±12.2)	0.37*
Functional bladder volume, ml	64.5 (±26.9)	67.4 (±19.0)	63.2 (±30.2)	0.72*
Diuria, *n*	25.3 (±7.0)	25.4 (±5.4)	25.2 (±7.8)	0.94*
Nocturia, *n*	8.2 (±3.3)		7.8 (±3.5)	0.39*
Postoperative results				
Functional bladder volume, ml	335.5 (±88.3)	381 (±113.5) 9.1 (±2.7)	323.3 (±84.3)	0.34*
Difference pre-/postoperative functional bladder volume, ml	+270.4 (±101.5)	+322.7 (±115.25)	+259.5 (±99.6)	0.34*
Diuria, *n*	5.7 (±2.0)	4.7 (±1.5)	5.9 (±2.1)	0.35*
Nocturia, *n*	1.4 (±1.0)	1.0 (±1.0)	1.5 (±1.0)	0.45*
Voluntary micturition, *n*	14 (53.8%)	2 (25%)	12 (66.7%)	0.036**/***
Catheterization, *n* (intermittent or suprapubic drainage)	9 (34.6%)	4 (50%)	5 (27.8%)	0.38**
Revision with urinary diversion, *n*	3 (11.5%)	2 (25%)	1 (5.6%)	0.63**
Pain recurrence, *n*	5 (19.2%)	2 (25%)	3 (16.7%)	0.62**

*Student’s *t* test

**Cross-tables Fisher’s exact test

***Statistically significant

The median follow-up period was 171 months (range: 89–264 months). No postoperative data could be collected after discharge in one case, and only record-based data could be obtained in three other cases.

Early postoperative complications requiring revision in 2 out of 27 cases (7.4%) were wound-healing impairment of the median laparotomy in one case and ileocolic anastomotic leakage in the other case. Late complications involved adhesive bowel obstruction with consecutive laparotomy and adhesiolysis in 1 of the 26 cases (3.8%). During the clinical course, 5 out of 26 patients (19.2%) had at least one episode of pyelonephritis, and 1 patient (3.8%) with concomitant ureteroneocystostomy developed poucho-ureteral reflux, recurrent pyelonephritis, and consecutive nephropathy. Bicarbonate was administered continuously for metabolic acidosis in 3 cases (11.5%). Poucholithiasis has not yet been reported. De novo stage 1 stress urinary incontinence was observed in 3 cases (11.5%) and stage 3 urine loss in 1 case (3.8%). In the latter case, a revision with secondary open Burch colposuspension led to hypercontinence, and the patient subsequently underwent cystectomy with ileal conduit placement after refusing ISC. None of the patients developed enuresis nocturna. No malignant degeneration was observed despite the long follow-up period.

### Functional results

In the initial postoperative phase, 24 out of 26 patients (92.3%) achieved a marked reduction in pain and urgency. Persistent symptoms in 2 patients (7.7%) necessitated early diversion within the first postoperative year. An ileocecal pouch with an appendix–umbilical stoma (Mainz pouch II) was created in one of them, and ureterosigmoidostomy was performed in the other. Five patients (19.2%) had recurrent pain and urgency after 3–9 years. Four of them described severe complaints with a reduced quality of life, and all required ISC. OnabotulinumtoxinA injections in the trigone did not reduce pain in 2 patients. Sacral neuromodulation was performed in 3 patients and successfully alleviated pain in 2 patients.

**Fig. 2:**
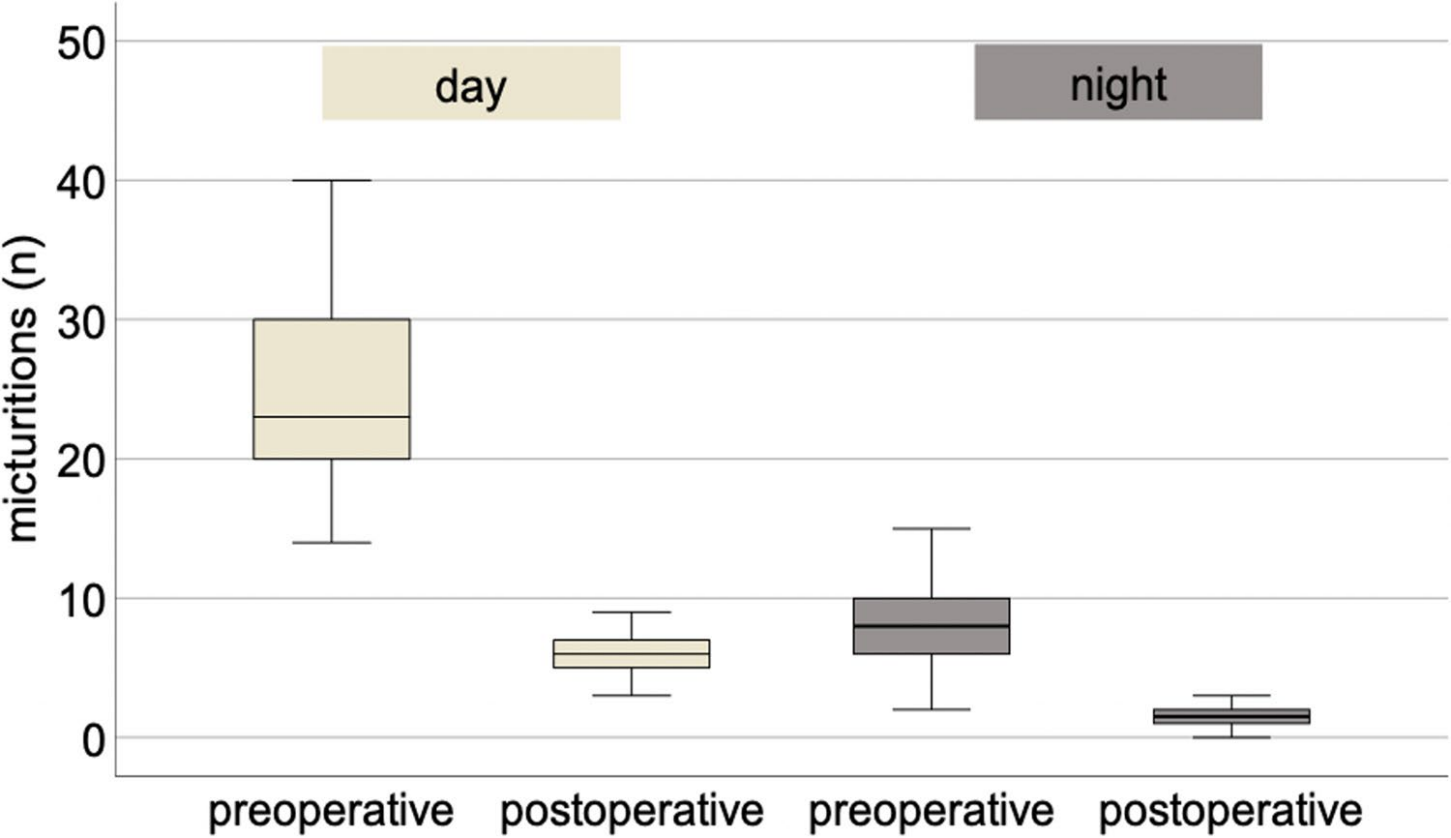
Pre- and postoperative micturitions day and night

**Fig. 3:**
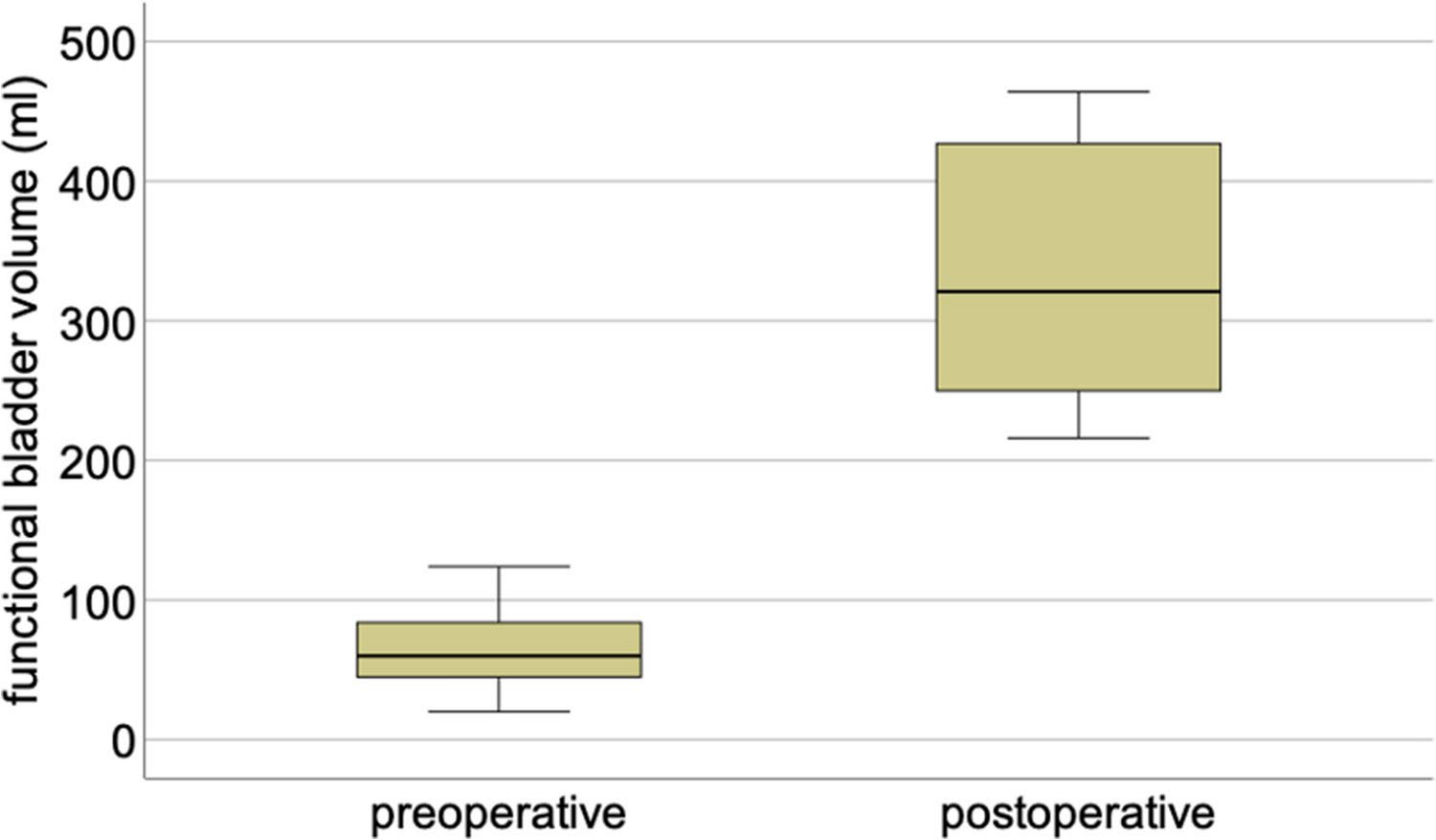
Pre- and postoperative functional bladder volume

Fourteen out of 26 patients (53.8%) emptied their augmented bladder voluntarily using both passive voiding and abdominal straining, whereas 8 (30.8%) underwent ISC. Diuria could be reduced to a mean micturition frequency of 6 and nocturia to 1.4 (range 0–3). The functional bladder volume increased to a mean of 265 ml in patients with sufficient voluntary micturition and to 374 ml in those with ISC (Figs. 2 and 3).

In one patient (4.3%) with a large residual urine volume, the Cystofix catheter remained in situ for 14.7 years. Diversion was performed in three cases (11.5%) for the above-mentioned reasons.

O’Leary Sant IC score was recorded at the time of last follow-up in 23 cases; the mean value was 12.7 out of 35 possible points. Patients responded to the PGI-I as follows: “very much better” in 15 cases (65.2%), “much better” in 7 (30.4%), and “a little better” in 1 case (4.3%). Neither of the patients with early revision and urinary diversion could be questioned regarding their satisfaction. All prospectively evaluated patients provided their consent to the operation.

In the univariate analysis, neither age at the time of surgery (*p* = 0.2) nor preoperative functional bladder volume (*p* = 0.2) correlated with satisfaction with the outcome of the intervention.

### Comparison of ileal and ileocecal augmentation

Mechanical bowel obstruction occurred in one patient, and intestinal anastomotic leakage occurred in another patient after ileocecal augmentation. Eight patients with ileal augmentation had no intestinal/surgical complications. The percentage of patients with sufficient voluntary micturition tended to be higher after ileocecal augmentation than after purely ileal augmentation (12 out of 18 [66.6%] vs 2 out of 8 [25%], *p* = 0.036). No differences were found in satisfaction with the interventions. The prospective evaluated PGI-I response was “much better” or “very much better” in 15 out of 16 cases (94.4%) for ileocecal and in 5 out of 7 cases for ileal augmentation (62.5%), but the results were not significantly different in the two groups (Student’s *t* test *p* = 0.2).

## Discussion

Even after a very long follow-up, which was longer than that reported elsewhere, we found a high level of patient satisfaction after supratrigonal cystectomy and augmentation. Diversion was only necessary in three cases and only within the first few years. None of the patients developed poucholithiasis or malignant degeneration during the long clinical course. Only one patient had relevant urinary incontinence. Urinary tract infections place little burden on patients. To the best of our knowledge, we are the first research group to demonstrate late recurrence of IC/BPS symptoms 3–8 years postoperatively in 19.2% of patients. Sacral neuromodulation was effective in 2 of these patients. It is already an established treatment alternative for IC/BPS, but has not previously been described in the therapy of late relapse after supratrigonal cystectomy and augmentation [9]. The cause of symptom relapse is generally assumed to be recurrent ulcerative IC/BPS in the trigone or residual detrusor [10–12]. The earlier assumption of an IC-type change even in the intestinal augmentation material has been abandoned because of histologically identical findings obtained by examining samples from patients who were complaint free after augmentation [13]. Thus, it seems important to remove as much of the detrusor as possible during supratrigonal cystectomy; the established periureteral margin was 1 cm or less in our procedure (Fig. 2). As already described by Rossberger et al. and Kontturi et al., the risk of adhesion-related vesicoureteral reflux should be kept in mind [11, 14], although symptomatic reflux in our cohort was observed in only one patient with synchronous ureteroneocystostomy during the primary intervention.

Establishing a correct indication is of major importance in the surgical treatment of ulcerative IC/BPS. This was done in our cohort by a urologist experienced in the treatment and diagnosis of IC. Particularly at low-volume centers, the decision regarding surgery should be made by a multidisciplinary team (urologist, pain therapist, urotherapist, and psychologist) [15]. The most important criterion is the clinical presentation of IC/BPS. Other research groups have already demonstrated a correlation of high satisfaction for patients with low bladder volume, as well as for those with preoperative detection of Hunner’s ulcers [14, 16, 17]. Univariate analysis of our data showed no correlation between preoperative bladder volume and outcome, although none of our patients had a functional bladder volume of more than 124 ml. Urethrocystoscopy revealed the clinical picture of severe ulcerative IC/BPS in all cases.

Only 7.7% of our patients underwent diversion owing to persistent complaints. All patients with late relapse and seriously reduced quality of life required regular self-catheterization. Therefore, this could be a major risk factor for recurrent complaints. Test catheterization prior to surgery can further reduce the failure rate by ruling out additional trigonalgia and predicting postoperative necessity [18]. Complete cystectomy should be considered if catheterization causes pain or is strictly refused, or if IC involves the trigone. Two research groups also obtained high satisfaction rates with this option [12, 19].

Hoy et al. and Steers found that voiding dysfunction and the need for ISC can be reduced by leaving the uterus or cervical stump in place. They reported that this increases the functional urethral length and avoids kinking, which is also reflected in the low prevalence of stress incontinence [20, 21]. The choice of the type and shape of the augmentation material also influences postoperative voiding function. In our analysis, patients with detubularized ileocecal augmentation material tended to achieve sufficient voluntary micturition more often. However, the use of the ileocecal segment is controversial. Proponents cite a longer mesentery, the retroperitoneal position of the intestinal anastomosis, and a lower incidence of metabolic acidosis [22–24], whereas other research groups contradict these statements and favor the use of ileum [18, 25, 26]. Apart from the arguments mentioned above, it should also be noted that only two complications in our cohort (mechanical bowel obstruction and anastomotic leakage) occurred after ileocecal augmentation. Detubularization of the augmentation material to increase capacity, improve compliance, and reduce the risk of enuresis nocturna is now undisputed, despite the fact that it lowers intravesical micturition pressures [27].

Osman et al. were indeed justified in criticizing the lack of valid assessment tools in most publications on the surgical therapy of IC/BPS [5]. Owing to the extremely long follow-up period, including patients who had already undergone surgery in 1991, our analysis involved only postoperative collection of the O’Leary Sant IC score, which was not validated until 1997 [28]. However, detailed analysis of pre- and postoperative bladder diaries and personal patient interviews has enabled us to make a valid statement regarding the great success of this operation.

### Conclusion

Supratrigonal cystectomy with substitution cystoplasty is a curative treatment option for low-capacity ulcerative IC/BPS in some patients. This intervention achieved very favorable long-term results in patients without trigonal involvement. The use of ileocecal augmentation material may increase the percentage of patients with voluntary micturition, although the choice of detubularized augmentation material (ileal/ileocecal) should be left to the surgeon. ISC is often necessary and has been identified as a risk factor for recurrent symptoms, although sacral neuromodulation offers a treatment alternative for this situation as well.
